# Overexpression of *AtTTP* Affects *ARF17* Expression and Leads to Male Sterility in Arabidopsis

**DOI:** 10.1371/journal.pone.0117317

**Published:** 2015-03-30

**Authors:** Zhi-Hao Shi, Cheng Zhang, Xiao-Feng Xu, Jun Zhu, Que Zhou, Li-Juan Ma, Jin Niu, Zhong-Nan Yang

**Affiliations:** College of Life and Environmental Sciences, Shanghai Normal University, Shanghai 200234, China; Wuhan University, CHINA

## Abstract

Callose synthesis is critical for the formation of the pollen wall pattern. CalS5 is thought to be the major synthethase for the callose wall. In the Arabidopsis anther, ARF17 regulates the expression of CalS5 and is the target of miR160. Plants expressing miR160-resistant ARF17 (35S:5mARF17 lines) with increased ARF17 mRNA levels display male sterility. Here we report a zinc finger family gene, AtTTP, which is involved in miR160 maturation and callose synthesis in Arabidopsis. AtTTP is expressed in microsporocytes, tetrads and tapetal cells in the anther. Over-expression lines of AtTTP (AtTTP-OE line) exhibited reduced male fertility. CalS5 expression was tremendously reduced and the tetrad callose wall became much thinner in the AtTTP-OE line. Northern blotting hybridization and quantitative RT-PCR analysis revealed that miR160 was decreased, while the expression of ARF17 was increased in the AtTTP-OE line. Based on these results, we propose that AtTTP associates with miR160 in order to regulate the ARF17 expression needed for callose synthesis and pollen wall formation.

## Introduction

The pollen wall is a specialized cellular structure which is important in angiosperm reproductive development[1]. It plays multiple functional roles, including pollen protection, hydration, pollen-stigma recognition and pollen tube elongation[2,3,4]. A large portion of sterile mutants are resulted from the defects in pollen wall. The pollen wall consists of exine, intine layers and tryphine (the pollen coat). The exine layer includes sexine (tectum and bacula) and nexine (the foot layer) in the outer layer of the pollen wall[5]. Intine, the inner layer, is comprised of cellulose, pectin and proteins. Tryphine layer is produced from the tapetum and deposited on the exine surface as well as in its cavities[3,6]. The pollen exine consists of sporopollenin secreted from the tapetum[7,8]. The correct pollen exine pattern depends on proper callose deposition and precise primexine formation during the early tetrad stages.

Callose (beta-1,3-glucan) is composed of polysaccharide materials. It behaves as a temporary cell wall around the plasma membrane during the tetrad stage. Callose synthase 5 (CalS5) is the major synthase of the callose wall[9,10]. In the case of *cals5* mutation, callose deposition is nearly completely absent. As a result, the baculae and tectum structure do not properly form and the tryphine is randomly deposited around the pollen surface[10]. UDP-glucose is reported to be a precursor for the synthesis of callose, and UDP-glucose pyrophosphorylase (UGPase) is responsible for the synthesis of UDP-glucose[11]. In the *atugp1 atugp2* double mutant, the callose around tetrads cannot be synthesized[12]. In *no primexine and plasma membrane undulation (npu)*, the primexine layer is absent and the callose in the tetrads is reduced, resulting in an aberrant pollen exine wall[13]. In the case of *cyclin-dependent protein kinase g1 (cdkg1)* mutation, callose synthesis is also impaired. CDKG1 regulates the pre-mRNA splicing of *CalS5* for pollen wall formation[14]. All of these mutants show that normal callose synthesis and deposition are important for pollen wall formation.

Several different microRNAs have been reported to be involved in plant reproductive development[15,16]. The over-expression of miR167 in Arabidopsis leads to both male and female reproduction defects[17,18]. *Trans-acting siRNA3 (TAS3)* is involved in the development of leaves and floral organs by cleavage of *AUXIN RESPONSE FACTOR3 (ARF3/ETT)*[19,20,21]. *Floral organs in carpels (foc)*, with a *Ds* transposon insertion in *miR160a*, exhibits a variety of defective phenotypes, including male sterility[22]. *ARF17* is the cleavage target of miR160. Plants expressing miR160-resistant ARF17 (*35S*:*5mARF17* lines) have an increased *ARF17* mRNA level and male sterility[23]. However, the knockout of *ARF17* also results in a male sterile phenotype with abnormal callose synthesis and primexine formation during anther development. In addition, ARF17 directly binds to the *CalS5* promoter to regulate its expression in callose synthesis[24].

TRISTETRAPROLINE (TTP) proteins are CCCH zinc finger proteins. In humans, the hTTP, miR16 and Ago/eiF2C family members form an RNA-induced silencing complex (RISC). This complex binds the AU-rich region of *alpha-TNF* and degrades its mRNA[25,26]. In Arabidopsis, At1g68200 (*AtTTP*) encodes a CCCH zinc finger protein which is the ortholog of hTTP. In this work, we investigated the function of *AtTTP* through transgenic over-expression lines. The results suggest that *AtTTP* is involved in miRNA maturation and pollen wall pattern formation in *Arabidopsis*.

## Results

### Phylogenetic relationship among the hTTP orthologs

**Fig 1:**
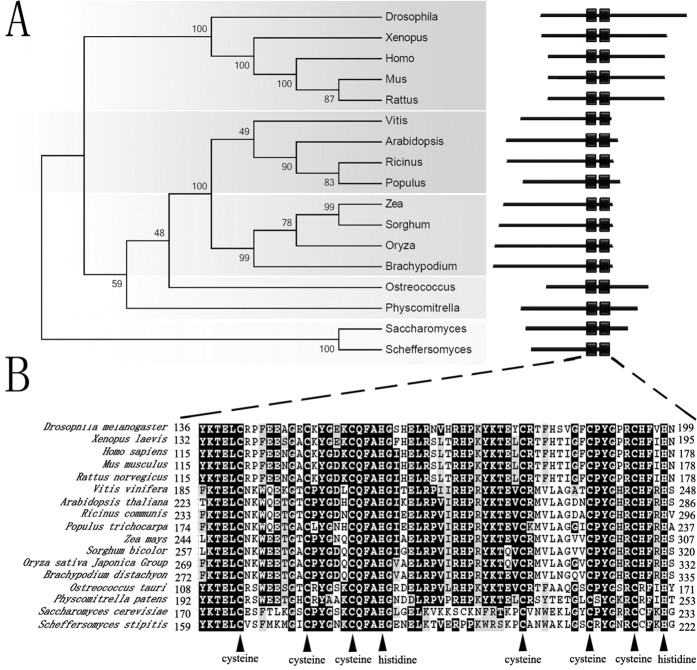
Phylogenetic analysis of *AtTTP* and orthologous proteins. (A) Unrooted phylogenetic tree of NPU and its orthologous proteins. The protein sequences of *AtTTP* and its orthologs were analyzed with the neighbor-joining method by MEGA5.05 software. The numbers at the nodes represent the percentage bootstrap values based on 1,000 replications. The CCCH domains were predicted by the Pfam 26.0 tool online. The protein sequence files are as follows: Drosophila: NP_511141.2; Xenopus: NP_001080610.1; Homo: NP_004917.2; Mus: NP_031590.1; Rattus: NP_058868.1; Vitis: XP_002281139.1; Arabidopsis: NP_176987.1; Ricinus: XP_002526299.1; Populus: POPTR_0010s12860.1; Zea: NP_001148404.1; Sorghum: XP_002440301.1; Oryza: NP_001056400.1; Brachypodium: XP_003569444.1; Ostreococcus: XP_003078184.1; Physcomitrella: XP_001783282.1; Saccharomyces: NP_013237.1; Scheffersomyces: XP_001385679.1. (B) Multiple alignments of *AtTTP* and its orthologs. Black triangles, the critical CCCH zinc finger residues: Cysteine and histidine.

To understand how the CCCH zinc finger genes affect the reproductive growth in plants, we searched for orthologs of the hTTP family proteins in Arabidopsis. The phylogenic relationship analysis showed that AtTTP was an ortholog of hTTP in Arabidopsis. The evolutionary relationship of these members is identical to that of species (Fig. 1A). AtTTP is closest to the orthologs of *Vitis, Ricinus* and *Populus* which are all dicots (Fig. 1A). According to the Pfam database, all of these proteins are conserved and contain two CCCH zinc finger domains (Fig. 1B). The characteristics of the CCCH zinc finger domains include C-X_8_-C-X_5_-C-X_3_-H and a linker of 18 residues that is located between the two zinc finger domains. The N-terminus of zinc finger domains contains a highly conserved sequence, KTEL (Fig. 1B).

### The expression pattern of *AtTTP*

**Fig 2:**
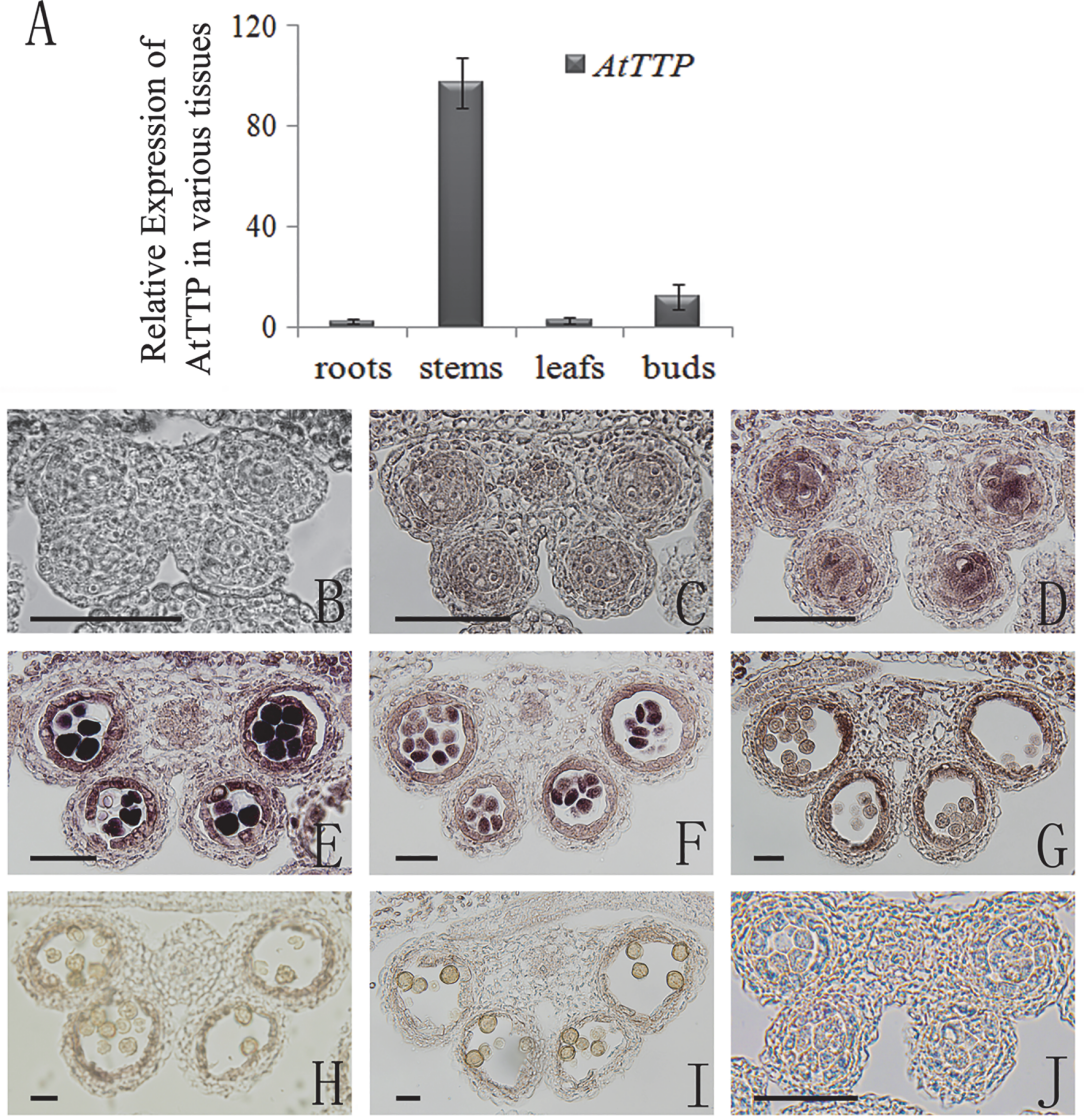
Expression analysis of *AtTTP*. (A) qRT-PCR analysis of RNA isolated from various tissues including roots, stems, leaves, and buds. Each expression level was normalized to that of *TUBULIN*. The data and errors bars are representative of 3 replicates. (B) *In situ* hybridization of the *AtTTP* transcript in a stage 4 anther with an antisense probe. (C) *In situ* hybridization of the *AtTTP* transcript in a stage 5 anther with an antisense probe. (D) *In situ* hybridization of the *AtTTP* transcript in an earlier stage 6 anther with an antisense probe. (E) *In situ* hybridization of the *AtTTP* transcript in a later stage 6 anther with an antisense probe. (F) *In situ* hybridization of the *AtTTP* transcript in a stage 7 anther with an antisense probe. (G) *In situ* hybridization of the *AtTTP* transcript in a stage 8 anther with an antisense probe. (H) *In situ* hybridization of the *AtTTP* transcript in a stage 9 anther with an antisense probe. (I) *In situ* hybridization of the *AtTTP* transcript in a stage 10 anther with an antisense probe. (J) *In situ* hybridization of the *AtTTP* transcript in an earlier stage 6 anther with an sense probe. Bars = 10 μm.

To characterize the expression pattern of *AtTTP*, we analyzed its expression in different tissues by quantitative reverse transcription (RT)-PCR. The results showed that *AtTTP* is expressed in stems and buds, with a weak expression in roots and leaves (Fig. 2A). Previous reports have suggested that *AtTTP* expression is markedly decreased in the *excess microsporocytes1 (ems1)/extrasporogenous cells (exs)* and *sporocyteless (spl)/nozzle(nzz)* mutant[27]. To elucidate the details of the expression pattern of *AtTTP* during anther development, we performed RNA *in situ* hybridization. The transcript signal of *AtTTP* was initially detected at anther development stage 5 (Fig. 2B-D) and reached a peak in microsporocytes (meiocytes) and tapetal cells at stage 6 (Fig. 2E). A strong signal was also detected in tetrads and tapetal cells at stage 7 (Fig. 2F), and the signal was sharply decreased at later stages (Fig. 2G-I). These results suggest that *AtTTP* may have a role in anther development.

During anther development, there is a genetic pathway (DYT1-TDF1-AMS-MYB103-MS1) downstream of *SPL/NZZ* and *EMS1/EXS* that is involved in tapetum development and function[28]. To understand the genetic relationship between *AtTTP* and this pathway, we analyzed the expression of *AtTTP* in *dyt1* and *tdf1* mutants by qRT-PCR. The *AtTTP* expression level in *dyt1* and *tdf1* was much higher than in the wild-type (Figure A in S1 File). To further elucidate the regulation of *AtTTP*, RNA *in situ* hybridization in the *dyt1* or *tdf1* background was performed. The *AtTTP* signal was detected in the microsporocytes and tetrads of these mutants (Figure A in S1 File). However, the signal could not be detected in the tapetal cells, suggesting that the aberrant tapetal cells impacted *AtTTP* expression (Figure A in S1 File).

### Over-expression of *AtTTP* reduced male fertility in Arabidopsis

**Fig 3:**
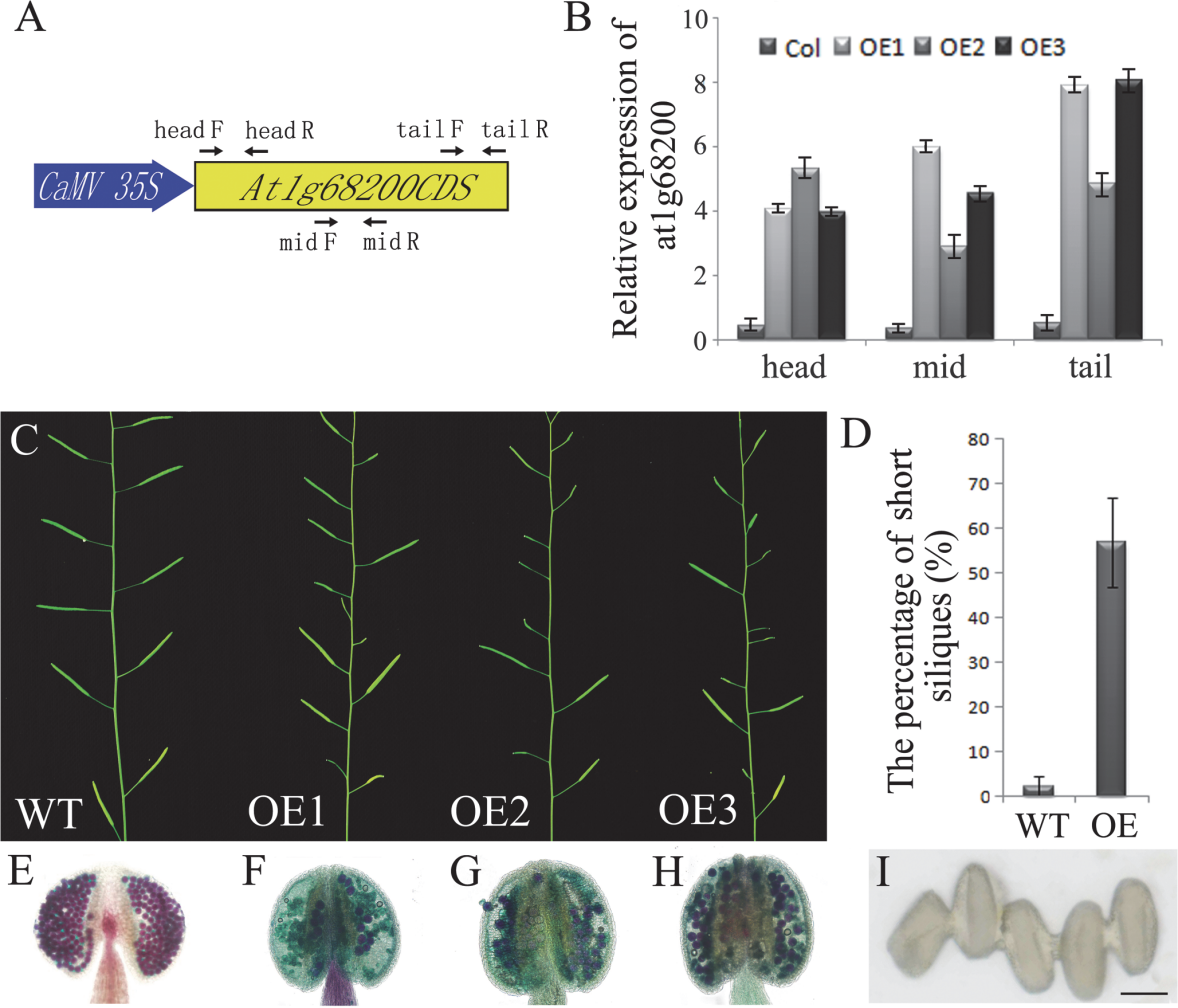
Characterization of *AtTTP*-OE line (A) Structure of the *AtTTP* over-expressing gene. The primers of head, mid and tail are used for real-time PCR amplification. (B) Real-time PCR analysis of *AtTTP* in wild-type and *OE1-3* mutant floral buds. The primer positions were shown in Fig. 3A. Each expression level was normalized to that of *TUBULIN*. The data and errors bars are representative of 3 replicates. (C) Comparison of reproductive development in 40-d-old wild-type and *OE1-3* mutant plants. (D) The percentage of short siliques in the wild-type and *OE1-3* mutant plants. (E) Alexander staining of the wild-type anther. (F) Alexander staining of the *OE1* mutant anther. (G) Alexander staining of the *OE2* mutant anther. (H) Alexander staining of the *OE3* mutant anther. (I) Adhered pollen from the *OE3* lines under light microscopy. Bars = 10 μm.

To further study the gene function of *AtTTP*, we identified two T-DNA tagged lines (Figure C in S1 File). In Salk_045897 line, the T-DNA was inserted into the first exon of *AtTTP*. The transcripts of *AtTTP* were disrupted (Figure C in S1 File). The phenotype of this line is similar to the wild type without any fertility defect. In Salk_065040 line, the T-DNA was inserted into the second exon. This line showed male sterility. We could not amplify the transcripts across the T-DNA insertion through PCR, which indicates that the open reading frame was interrupted (Figure C in S1 File). However, when the primers downstream of the T-DNA insertion were used to perform PCR, the products were significantly increased in contrast with that of the wild type (Figure C in S1 File). The expression of 5’ end of the *AtTTP* (upstream of the T-DNA insertion site) was not affected in the T-DNA line. This suggests that the internal 35S promoter in T-DNA might activate the expression of the 3’ end of the *AtTTP* open reading frame. Based on these results, we predicted that the extra-transcripts of *AtTTP* caused the male fertility phenotype in Salk_065040. We then constructed *CaMV 35S*: *AtTTP CDS* for the over-expression analysis (Fig. 3A) and obtained five transgenic lines. All the five T1 transgenic lines showed reduced fertility in various degrees. All the T1 progenies with reduced fertility also contained the transgene. Three of the five lines were chosen for further analysis. All these three lines exhibit increased *AtTTP* expression and reduced seed sets (Fig. 3B-D). Alexander staining[29] was performed to analyze the male fertility defects in the *AtTTP*-OE line. As shown in Fig. 3, the wild-type pollen grains were stained purple, indicating that they developed properly (Fig. 3E). In contrast, most of the aborted pollen grains were stained green (Fig. 3F-H) and a few normal pollen grains adhered together in the anther (Fig. 3I). The Scanning Electron Microscopy (SEM) results also indicate that the pollen grains in *AtTTP*-OE lines adhered to each other (Figure B in S1 File).

### Callose synthesis is dramatically reduced in *AtTTP*-OE lines

**Fig 4:**
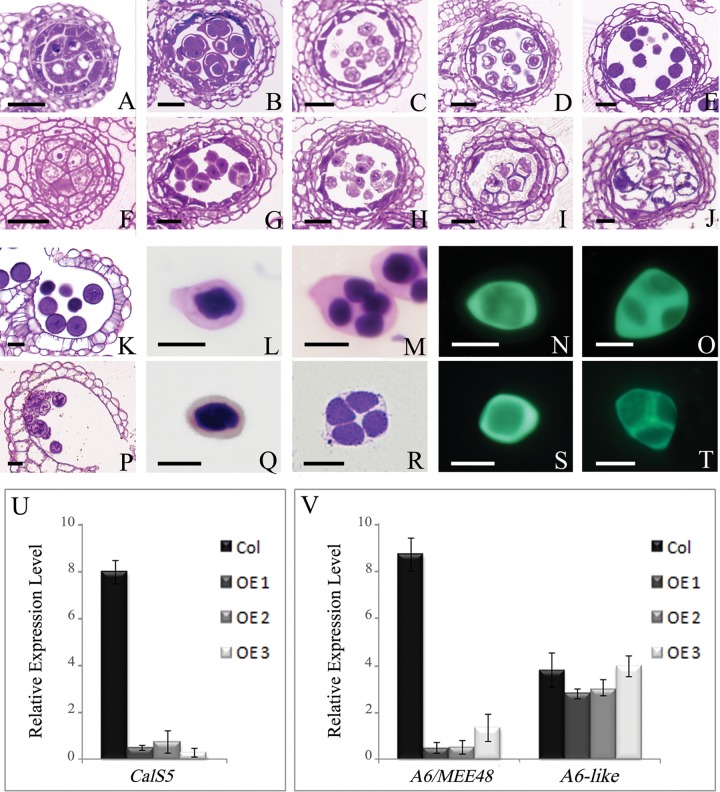
The anther development, callose wall, and expression analyses of *CalS5*, *A6* and *A6-like* in the wild-type and *AtTTP*-OE line. (A and F) Anthers at stage 6. The *AtTTP*-OE microsporocytes (F) are apparently closely compacted compared with those of the wild-type (A). (B and G) Anthers at stage 7. Wild-type tetrads are surrounded with callose (B), whereas the tetrads lack callose in the *AtTTP*-OE line (G). (C and H) Anthers at stage 8. Wild-type microspores are released from the tetrads (C), whereas microspores were still adherent in the *AtTTP*-OE line (H). (D and I) Anthers at stage 9. The microspores have begun to degenerate and still have not been released from the tetrads in the *AtTTP*-OE line (F). (E and J) Anthers at stage 10. The microspores are disintegrated in the *AtTTP*-OE line (J). (K and P) Anthers at stage 12. Remnants of microspores and less abnormal microspores were observed in the *AtTTP*-OE anther locule (P). (L, Q, N, S) The callose wall in the *AtTTP*-OE line was not obviously different compared with that of the wild-type. (M, R, O, T) The callose wall in the *AtTTP*-OE line was thinner around the tetrads compared with that of the wild-type. (U) Real-time PCR analysis of *CalS5*. Each expression level was normalized to that of *TUBULIN*. The data and errors bars are representative of 3 replicates. (V) Real-time PCR analysis of *A6* and *A6-like*. Each expression level was normalized to that of *TUBULIN*. The data and errors bars are representative of 3 replicates. Bars = 10 μm.

To elucidate the anther development defects in detail, cross sectioning of the *AtTTP*-OE1 line was performed. Based on a previous report[30], anther development is divided into 14 well-ordered stages in Arabidopsis. Our results showed that the anther development in *AtTTP*-OE line was similar to that of the wild-type from stage 1 to stage 6 (Fig. 4A and F). However, at stage7, the callose around tetrads in the *AtTTP*-OE line was thinner than that of the wild-type (Fig. 4 B and G). At stage 8, the angular microspores were released from the tetrads in the wild-type (Fig. 4C). In contrast, microspores were observed within the tetrads in the *AtTTP*-OE line (Fig. 4H). At stage 9, an exine wall was observed around the microspores and vacuolation was evident in the wild-type (Fig. 4D). In the *AtTTP*-OE line, the microspores still adhered together and begun to degenerate (Fig. 4I). During stages 10 to 12 in the wild type, microspores formed a mature pollen wall and were released from the dehisced anther (Fig. 4E and K). The microspores in the *AtTTP*-OE line were expanded, integrated together and eventually aborted (Fig. 4J and P). During the course of anther development, no obvious difference was observed in tapetum development in the *AtTTP*-OE line.

To further examine the tetrad defect in the *AtTTP*-OE line, aniline blue and toluidine blue were used to stain the microsporocytes and tetrads. The callose around microsporocytes was similar in the wild-type and *AtTTP*-OE line (Fig. 4L, Q and N, S), indicating that the callose synthesis in the microsporocytes may not be affected. However, at the tetrad stage, the callose fluorescent signal in the *AtTTP*-OE line was much weaker than in the wild-type (Fig. 4O and T). The callose wall of *AtTTP*-OE line was much thinner than that of the wild type (Fig. 4M and R). We then analyzed the expression of the *CalS5* gene in the *AtTTP*-OE line, a gene which was reportedly responsible for callose synthesis[9,10]. In the *AtTTP*-OE line, *CalS5* was down-regulated approximately 9 fold less than that in the wild-type, as shown by qRT-PCR analysis (Fig. 4U). These results suggest that the over-expression of *AtTTP* does exert an effect on the *CalS5* expression needed for callose synthesis in anther development. We further tested the expression of the *A6* and *A6-like* genes, which are reportedly responsible for callose dissolution[31]. In the *AtTTP*-OE line, *A6* expression was reduced while *A6-like* genes exhibited no obvious change (Fig. 4V). We also performed the cytological characterization of Salk_065040. The T-DNA line exhibited similar but severer phenotype to that of the OE line (Figure D and E in S1 File), which was in accordance with the results shown in [32]. These results indicate that *AtTTP* plays a role on callose dissolution.

### miR160 was decreased in the *AtTTP*-OE line

**Fig 5:**
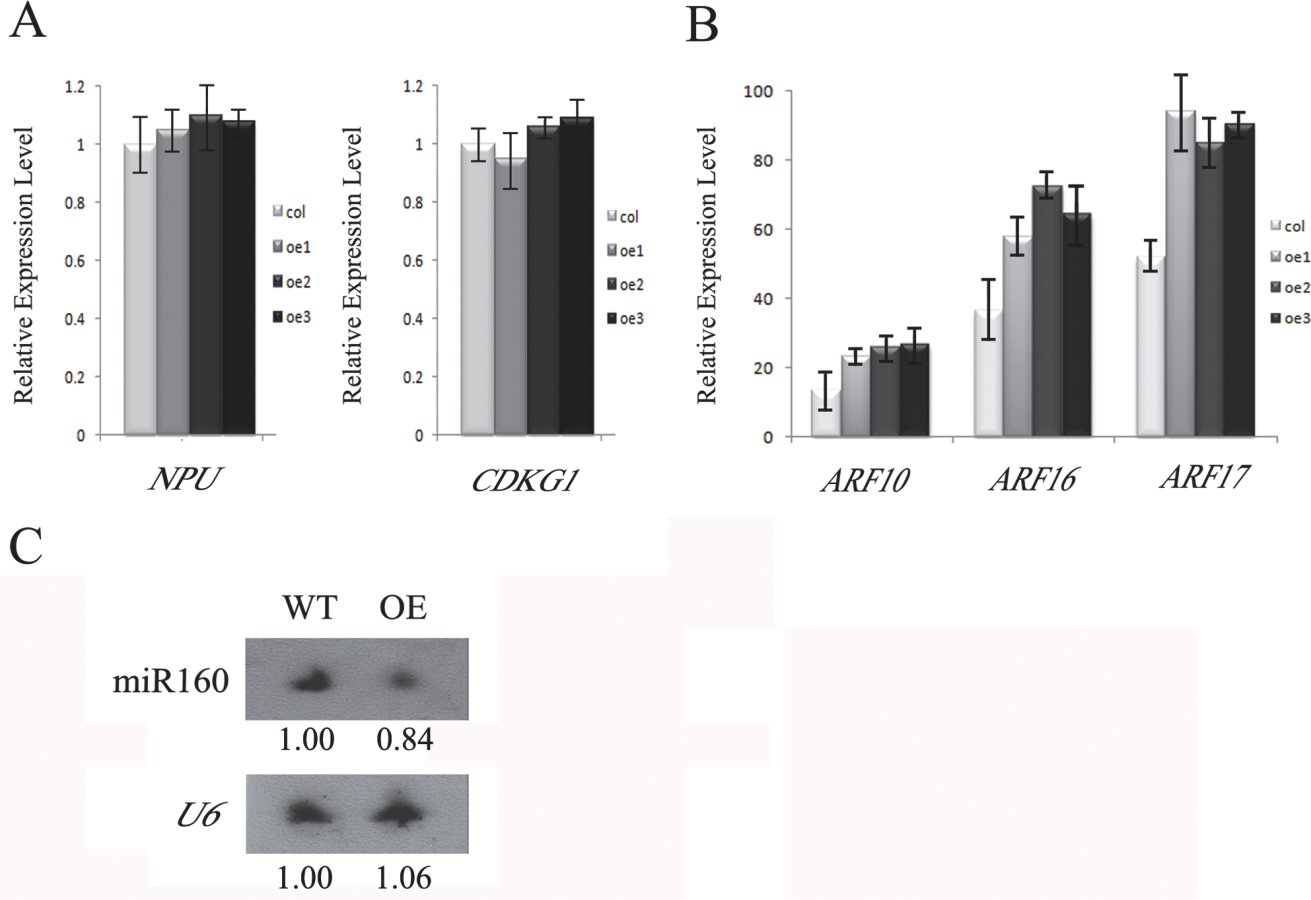
Expression analysis of *NPU*, *CDKG1*, *ARFs* and miRNAs in the AtTTP-OE line. (A) Real-time PCR analysis of *NPU* and *CDKG1* in the *AtTTP*-OE line. Each expression level was normalized to that of *TUBULIN*. The data and errors bars are representative of 3 replicates. (B) Real-time PCR analysis of *ARF10, ARF16* and *ARF17* in the *AtTTP*-OE line. Each expression level was normalized to that of *TUBULIN*. The data and errors bars are representative of 3 replicates. (C) Northern blotting hybridization of mature miR160 in the *AtTTP*-OE line.

To understand how *AtTTP* affects the expression of *CalS5*, we analyzed the expression of *NPU, CDKG1* and *ARF17*, which were shown to affect the expression of *CalS5*[13,24]. Quantitative RT-PCR analysis showed that the expression of *NPU* and *CDKG1* was unaffected (Fig. 5A), while *ARF17* expression was increased in the *AtTTP*-OE line (Fig. 5B). Previous investigation showed that *ARF10, ARF16* and *ARF17* are cleavage targets of miR160. In transgenic plants expressing miR160-resistant ARF17 (*35S*:*5mARF17* lines), the *ARF17* mRNA level was increased and the transgenic lines exhibited male sterility[23]. In the *AtTTP*-OE line, the expression level of *ARF10* and *ARF16* was also increased (Fig. 5B). The increased expression level in all the three miR160 target genes suggests that miR160 is affected in the *AtTTP*-OE line. Therefore, we analyzed the expression of miR160 by Northern blotting hybridization. The results showed that the mature miR160 was indeed reduced in the *AtTTP*-OE line (Fig. 5C).

## Discussion

### Over-expression of *AtTTP* affects both callose wall synthesis and the pollen wall pattern

Callose synthesis, plasma membrane undulation and primexine deposition are essential for building the properly sculptured exine needed for the pollen wall pattern[13,33,34]. CalS5 is the major enzyme for callose wall synthesis. The absence of peripheral callose wall around *cals5* tetrads has been shown to exert an effect on the pollen wall pattern[10]. Several genes affecting *CalS5* expression and pollen wall pattern have been reported. CDKG1 is associated with the splicesome for the pre-mRNA splicing of *CalS5*[14]. *NPU* encodes an unknown membrane protein which affects *CalS5* expression and callose synthesis as well as plasma membrane undulation and primexine deposition[13]. Based on the analysis of Salk_045897 (Figure C in S1 File), the disruption of *AtTTP* transcripts could not affect callose deposition and male fertility. *AtTTP* belongs to the CCCH zinc finger family which contains 68 members in *Arabidopsis*. Probably other members in this family play a redundant function with *AtTTP* for callose synthesis. In Salk_065040 line, the activated C-terminal may interfere in the expression of *AtTTP* and its homologs which leads to the defect of callose synthesis and plant fertility. In the *AtTTP*-OE line, the over-expression of *AtTTP* affected both *Cals5* expression and callose synthesis (Fig. 4). The abnormal pollen wall pattern in the *AtTTP*-OE line displayed defective sporopollenin deposition (Figure A in S1 File). The collapsed pollen wall was similar to that of *cals5, npu* and *cdkg1*[10,13,14]. *AtTTP* is a novel factor that modulates callose wall synthesis and the pollen wall pattern.

### miR160-ARF17 modulates callose wall synthesis

It is reported that *ARF10, ARF16* and *ARF17* are the cleavage targets of miR160[23,35,36,37]. The increased expression levels of *ARF17* in *35S*:*5mARF17* transgenic lines result in a male sterile phenotype. However, the amount of excess *ARF17* that leads to the male sterile phenotype is still unclear[23]. In this study, the over expression of *AtTTP* reduced the amount of mature miR160 in the anther, and the expression of *ARF10, ARF16* and *ARF17* were all increased (Fig. 5). These results support the notion that *ARF10, ARF16* and *ARF17* are the targets of miR160. In addition, because the mRNA level of *ARF17* is increased in both the *AtTTP*-OE and *35S*:*5mARF17* transgenic lines, this suggests that callose wall synthesis and pollen wall pattern were also affected in the *35S*:*5mARF17* transgenic lines as in the *AtTTP*-OE line. The *35S*:*5mARF17* transgenic lines resulted in a completely sterile mutant, whereas the knockout mutant *cals5* was only semi-sterile. This suggests that the over-expression of *ARF17* in the *35S*:*5mARF17* transgenic lines not only affect *CalS5* expression, but it may also affect other genes needed for anther development.

Recently, ARF17 was shown to directly bind to the promoter region of *CalS5* and thus regulate its expression in callose wall synthesis. The knock-out *ARF17* line displays complete male sterility along with reduced callose synthesis[24]. In both of the *AtTTP*-OE lines in this work (Fig. 3) and also *35S*:*5mARF17* transgenic lines[23], the expression level of *ARF17* was increased. However, these lines also exhibited defective male fertility. It was reported that miRNAs could up-regulate translation[38]. In the *35S*:*5mARF17* transgenic lines, miR160 cannot bind to *ARF17* because of the existing point mutations. This may affect *ARF17* translation and *35S*:*5mARF17* transgenic lines display a phenotype similar to the *arf17* knock-out mutant phenotype. In the *AtTTP*-OE line, the reduced level of miR160 may also affect the translation of *ARF17*, which in turn leads to defective pollen wall formation and male sterility. However, this is a subject that needs to be further investigated in future work.

### *AtTTP* may associate with miR160 to regulate *ARF17* expression

The CCCH zinc finger family members have been studied in various organisms. There are 68 CCCH family genes in *Arabidopsis*, 67 in rice and 91 in *Populus*[39]. Several members have been discovered to be involved in a variety of biological processes. For instance, HUA1 is involved in flower development[40], SUMNUS in seed germination[41], AtSZF1 and AtSZF2 in the salt stress response[42], and OsDOS in leaf senescence[43]. These five proteins are all localized in the nucleus. However, recently there have been reports of two novel CCCH zinc finger proteins, AtTZF1 and OsLIC, which are localized in both the nucleus and cytoplasm[44,45]. AtTZF1 may function as a nucleon-cytoplasm shuttling protein or as a functional unit of the processing-bodies (P-bodies) in the cytoplasm instead of a traditional transcription factor in the nucleus[44]. In humans, hTTP, miR16 and the Ago/eiF2C family members form an RNA-induced silencing complex (RISC) that binds the AU-rich region of *alpha-TNF* so as to degrade its mRNA[26,46]. AtTTP is an ortholog of hTTP in Arabidopsis that is located in the cytoplasm and is involved in the secondary cell wall formation, but its precise function is still unknown[47,48]. AtTTP has been predicted to be located in P-bodies[44,47]. P-bodies contain RNA-protein complexes involved in cytoplasmic RNA processing pathways and post-transcriptional regulation[49]. In this work, the over-expression of *AtTTP* affected the expression of *ARF17*, and the mature miR160 was reduced in the AtTTP-OE line. We propose that AtTTP associates with miR160 to regulate *ARF17* expression in P-bodies as a mean for hTTP to regulate the *alpha-TNF* expression. The over-expression of *AtTTP* may interfere in the RISC, which in turn affects the maturation of miR160 in the AtTTP-OE lines.

**Fig 6:**
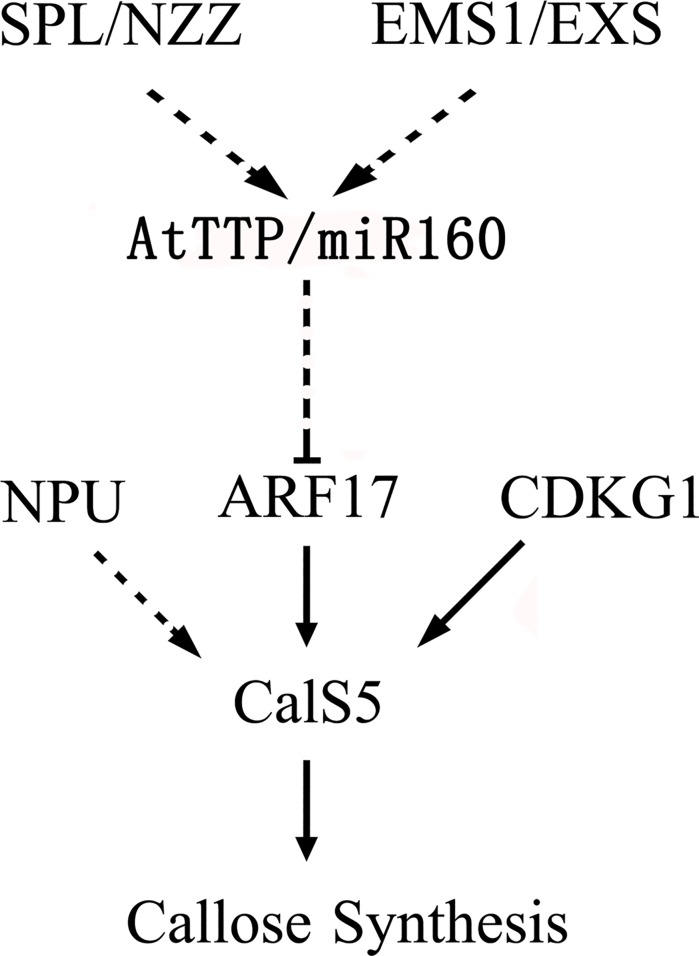
A putative regulation pathway in anther. Black arrow: direct regulation has been demonstrated; Dotted arrow: the regulatory mechanism remains unclear.

In the Arabidopsis anther, *SPL/NZZ* and *EMS1/EXS* are involved in cell differentiation patterns and early sporogenesis[50,51,52,53]. The decreased expression of *AtTTP* in both *spl/nzz* and *ems1/exs* suggests that it functions downstream of these two genes[27]. In this work, a functional analysis of *AtTTP* through the *AtTTP*-OE line has revealed the pathway AtTTP-miR160-ARF17-CalS5 in the Arabidopsis anther (Fig. 6). This pathway plays a regulatory role in callose synthesis and the pollen wall pattern.

## Materials and Methods

### Plant Materials

The Arabidopsis plants used in this study are from an ecotype with a Columbia-0 background. SALK_045897 was obtained from ABRC. Plants were grown under long-day conditions (16h of light/8h of dark) in an approximately 22°C growth room.

### Protein Alignment and Phylogenetic Analysis

Phylogenetic trees were constructed and tested by MEGA5.05 based on the neighbor-joining method. The CCCH domains were predicted by the Pfam 26.0 tool online (http://pfam.sanger.ac.uk/). The multiple sequence alignment of full-length protein sequences was performed using ClustalX 2.0 software and displayed using the BOXSHADE tool online (http://www.ch.embnet.org/software/BOX_doc.html).

### Expression Analysis

For expression analysis of *AtTTP*, RNA was extracted from roots, stems, leaves, and buds. The quantitative RT-PCR was used to assess the expression level of of *AtTTP* (At1g68200), using the primer sets as follows: qRT-F (5’- TATGTGCGAGGAGGAGGGAA-3’) and qRT-R (5’- CAGTCTTGTAACGGGGATGG-3’).

The DIG (for digoxigenin) RNA labeling kit (Roche) and PCR DIG probe synthesis kit (Roche) were used for the RNA in situ hybridization experiment. An *AtTTP*-specific cDNA fragment was amplified and cloned into the pSK vector. Antisense and sense DIG-labeled probes were prepared as described[54].

To assess the expression of *AtTTP* in the transgenic lines, Real-time PCR was used to amplify three fragments (head, mid and tail) of the *AtTTP* transcript, using the primer sets as follows: Head-F (5’-ACTCGCAACATAATGCAACAGC-3’) Head-R (5’-TCGTCGGCGTCATTACCTCC-3’) Mid-F (5’-CTAAGCCTGGGACTTGTGGTC-3’) Mid-R (5’-TGAGCGAACTGGCAATGGT-3’) Tail-F (5’-GCGAGGAGGAGGGAAGAA-3’) Tail-R (5’-GCGGATCACTGGACGGAG-3’).

### Plasmid Construction

A 927bp fragment for the *AtTTP* was amplified using polymerase chain reaction (PCR) with the cDNA from wild-type Col plants using gene-specific primers (5'-cc ggtacc ATGGAAAACAAAATCGCGCC-3' and 5'-cc gaattc TCATGTGATCAGCTTGAGGG-3'). This DNA fragment was cloned into a pMD18-T vector (TaKaRa, Japan), verified by sequencing, and inserted into the plant transformation vector pMON530 (Monsanto, USA) downstream of the *CaMV 35S* promoter. Then this plasmid was introduced into Columbia plants by Agrobacterium-mediated transformation.

### Phenotype Characterization and Microscopy

Plants were photographed with a Nikon digital camera (Coolpix 4500). Flower images were taken using an Olympus dissection microscope with an Olympus digital camera (BX51). Alexander solution was used as described[29]. Plant material for the semithin sections was prepared and embedded in Spurr’s resin, as previously described[55]. For SEM examination, fresh stamens and pollen grains were coated with 8 nm of gold and observed under a JSM-840 microscope (JEOL).

### Northern Blotting Hybridization and Real-Time PCR

For expression analysis of the miRNAs in the transgenic lines, RNA was extracted from buds of *AtTTP*-OX T2 generation and wild-type plants using TRizol (Invitrogen). Approximately 10 μg total RNA was electrophoresed in 15% PAGE gel and transferred onto a nylon membrane (Pall, Mexico, NM). The DNA-LNA mixed probe sequence against miR160 is 5’-TGGCATACAGGGAGCCAGGCA-3’ (EXIQON, Product no.: 42009-00). The DNA probe (sequences used) sequence against U6 is 5’-CTCGATTTATGCGTGTCATCCTTGC-3’. The probe labeling with digoxygenin, hybridization and chemiluminescent detection were carried out according to the Roche manual (Roche, Basal, Switzerland, http://www.roche.com). The primers of *ARF10, ARF16* and *ARF17* were designed according to a previous report[22].

## Supporting Information

S1 File Figure A, Expression analysis of AtTTP in dyt1 and tdf1. **Figure B**, SEM examination of dehiscent anthers and pollen grains of the wild-type (A, D, G and J), strong phenotype (B, E, H and K) and weak phenotype (C, F, I and L) in the *AtTTP*-OE line. **Figure C**, The T-DNA insertion and expression analysis of *AtTTP*. **Figure D**, The phenotype characterization of Salk_065040. **Figure E**, The TEM observation of pollen mother cells, tetrads and pollen grains of Salk_065040.
(PDF)
