# Supplementary material for: Overexpression of AtTTP Affects ARF17 Expression and Leads to Male Sterility in Arabidopsis
Source: PLoS One. 2015 Mar 30;10(3):e0117317. doi: 10.1371/journal.pone.0117317 (PMC4378849; doi:10.1371/journal.pone.0117317)
Supplement: S1 File — Figure B, SEM examination of dehiscent anthers and pollen grains of the wild-type (A, D, G and J), strong phenotype (B, E, H and K) and weak phenotype (C, F, I and L) in the AtTTP-OE line. Figure C, The T-DNA insertion and expression analysis of AtTTP. Figure D, The phenotype characterization of Salk_065040. Figure E, The TEM observation of pollen mother cells, tetrads and pollen grains of Salk_065040. (PDF) [file pone.0117317.s001.pdf]

**Figure A**

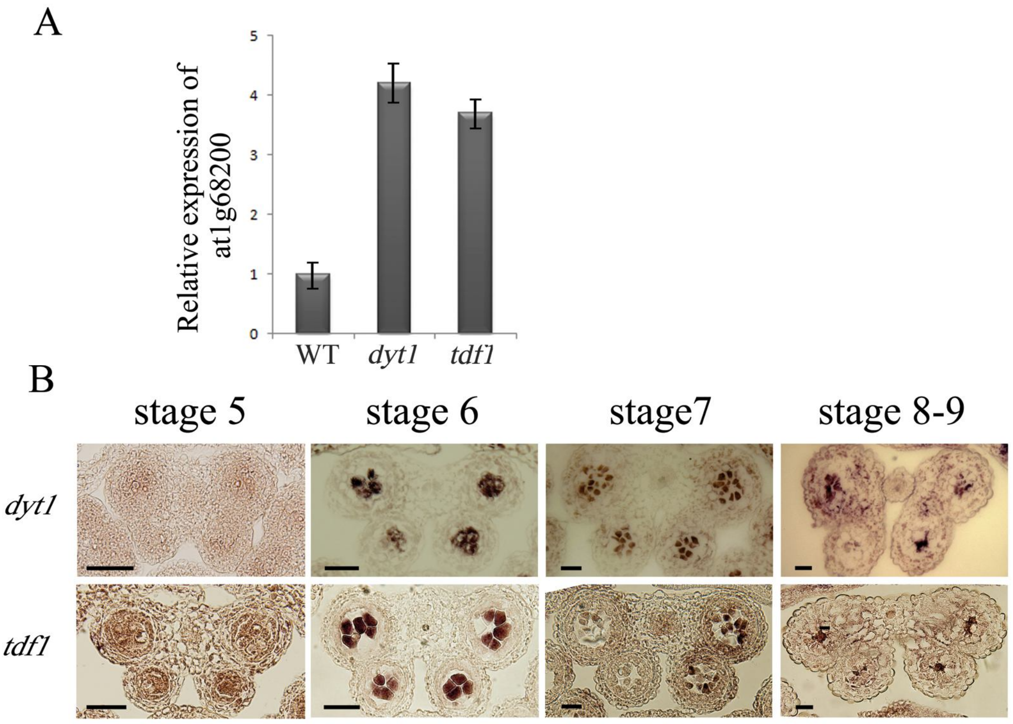


(A) Real-time PCR analysis of *AtTTP* in in *dyt1* and *tdf1* floral buds. Each expression level was normalized to that of *TUBULIN*.

(B) *In situ* hybridization of the *AtTTP* transcript in the *dyt1* and *tdf1* anther.

Bars = 10 μm

**Figure B**

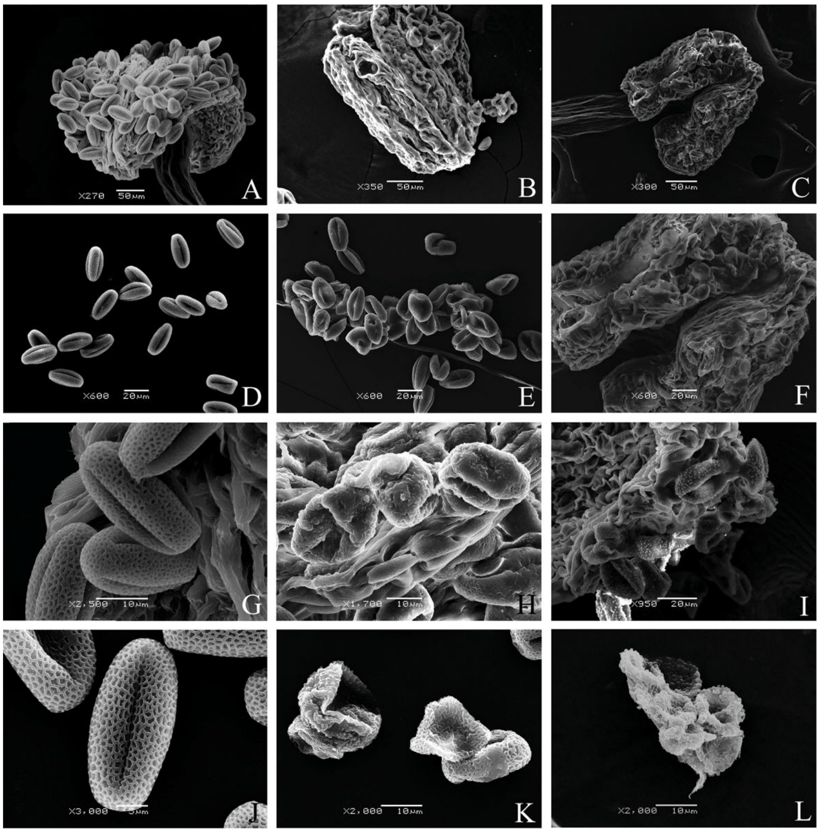

(A, B and C) Dehiscent anthers. Wild-type anther contained numerous pollen grains, whereas most of the *AtTTP*-OE pollen grains were degenerated and adhered to the anther.

(D, E and F) Pollen grains. Wild-type pollen grains were separated from each other, whereas *AtTTP*-OE pollen grains adhered to each other (E) and even were inseparable from the anther (F).

(G, H and I) Pollen grains on the dehiscent anther.

(J, K and L) Pollen exine pattern. Wild-type pollen grains have a regular reticulate exine pattern, whereas ruptured *AtTTP*-OE pollen grains exhibit a spotted exine pattern.

**Figure C**

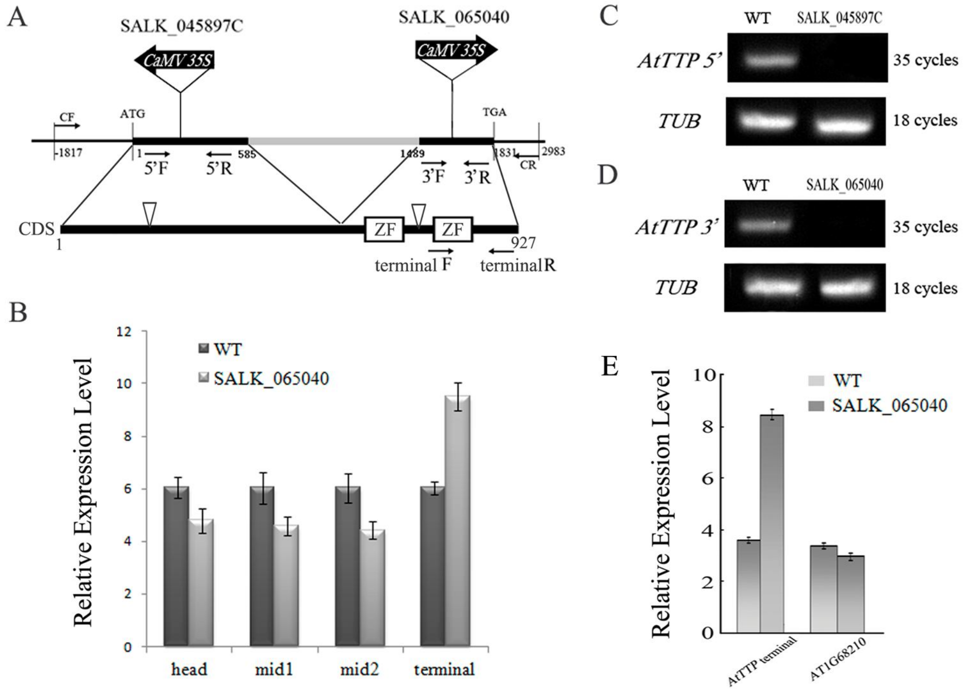


A, the T-DNA insertion of SALK_045897 and the name of primers used in the expression analysis of AtTTP gene.

B, the expression analysis of different part of *AtTTP* gene in SALK_065040. Head, the 109$^{th}$ to 356$^{th}$ bp of *AtTTP* CDS sequence. Mid1, the 551$^{st}$ to 740$^{th}$ bp of *AtTTP* CDS sequence. Mid2, the 436$^{th}$ to 615$^{th}$ bp of *AtTTP* CDS sequence.

C, the expression analysis of *AtTTP* gene in both wild type and SALK_045897.

D, the expression analysis of C-terminal of *AtTTP* gene in both wild type and Salk_065040.

E, the C-terminal over-expression of *AtTTP* and At1g68210 in wild type and Salk_065040.

**Figure D**

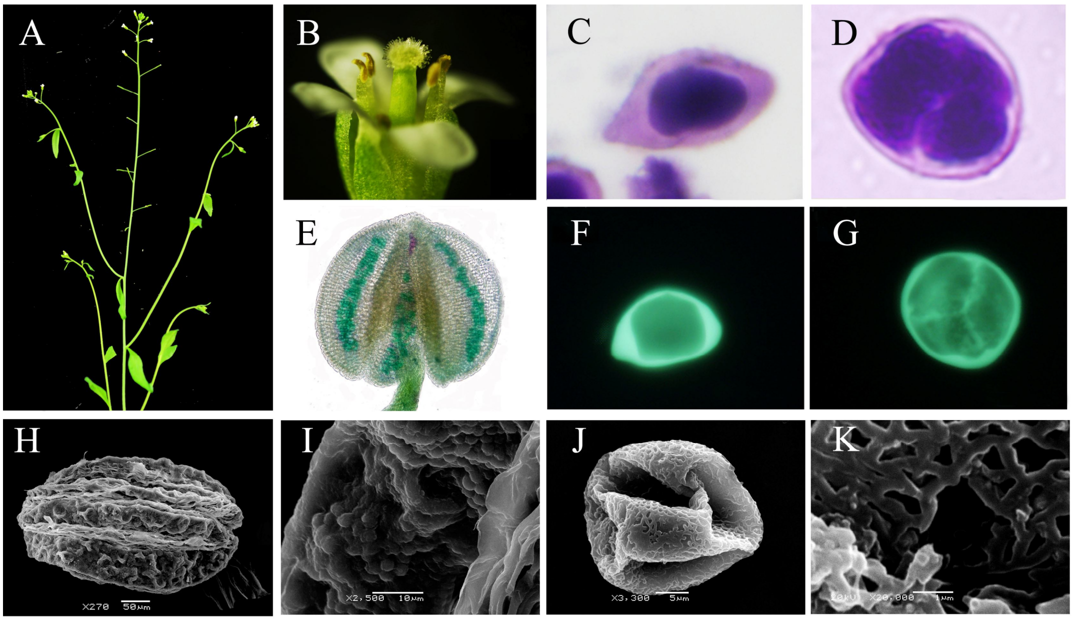


A, Salk_065040 showed male sterility.

B, the flower of Salk_065040

C and D, the pollen mother cells and tetrads of Salk_065040

E, pollen staining of Salk_065040

F and G, callose staining of pollen mother cells and tetrads of Salk_065040

H to K, the SEM observation of Salk_065040 pollen grains

**Figure E**

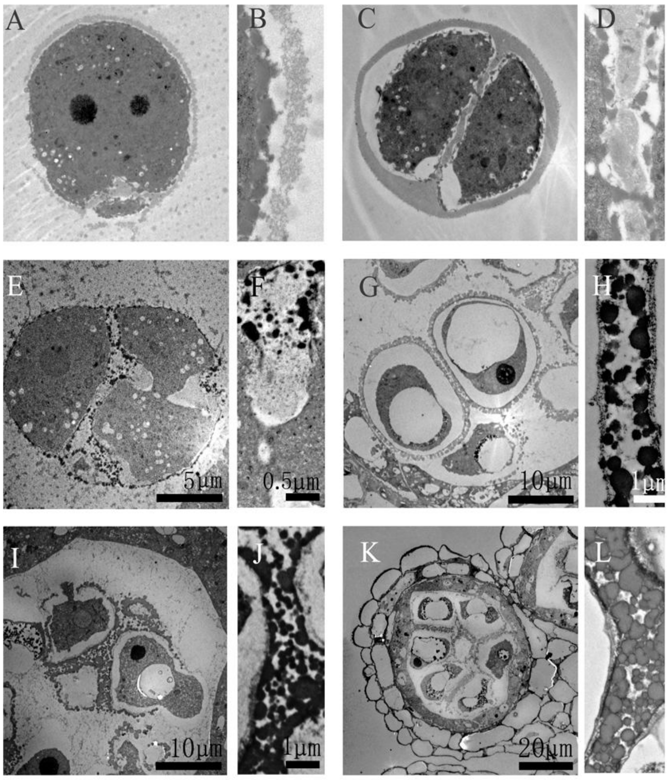


A and B, pollen mother cell

C to F, tetrads

G to I, adhered microspores

J to L, adhered microspores and abnormal sporopollenin deposition
